# Sphingosine kinase 1 mediates head & neck squamous cell carcinoma invasion through sphingosine 1-phosphate receptor 1

**DOI:** 10.1186/s12935-014-0076-x

**Published:** 2014-08-29

**Authors:** Paulette M Tamashiro, Hideki Furuya, Yoshiko Shimizu, Toshihiko Kawamori

**Affiliations:** Cancer Biology Program, University of Hawaii Cancer Center, 701 Ilalo Street, Honolulu, HI 96813 USA; Department of Molecular Biosciences and Bioengineering, University of Hawaii at Manoa, Honolulu, HI 96818 USA; Department of Pathology, Ichinomiya Nishi Hospital, 1 Hira, Kaimei, Ichinomiya, Aichi Pref., 494-0001 Japan; Clinical and Translational Research Program, University of Hawaii Cancer Center, 701 Ilalo Street, Honolulu, HI 96813 USA

**Keywords:** Sphingosine kinase1, Invasion, HNSCC, EGFR, STAT3, Sphingosine 1-phosphate, Sphingosine 1-phosphate receptor 1, IL-6, SCC-25 cells

## Abstract

**Background:**

Head and neck squamous cell carcinoma (HNSCC) is characterized by aggressive loco-regional invasion. Sphingosine kinase1 (SphK1), an enzyme in sphingolipid metabolism, is emerging as a key player in HNSCC pathology. The observation that SphK1 is overexpressed in all HNSCC stages and is associated with depth of tumor invasion, metastasis and clinical failure underscores the importance of SphK1 in HNSCC pathology. Still, the mechanisms underlying SphK1 regulation of invasion have not been delineated. Therefore, we sought to mechanistically describe how SphK1 regulates invasion in HNSCC.

**Methods:**

Invasion assays were used to measure invasive ability of SphK1 overexpressing human tongue squamous cell carcinoma (SCC-25 cells). Western blotting, quantitative qPCR, ELISA and zymography were used to measure the effect of SphK1 and sphingosine 1-phoshate receptor 1 (S1P_1_) on invasion measures, MMP-2/9, E-cadherin, EGFR, IL-6/STAT3, in SCC-25 cells.

**Results:**

SphK1 expression is elevated in cells with an invasive phenotype as compared to non-invasive phenotype. We show SphK1 overexpression increased EGF-induced EGFR/ERK and AKT activity, increased matrix metalloproteinase (MMP)-2/9 mRNA and reduced E-cadherin. SphK1 overexpression also increased IL-6 concentration and EGF-induced STAT3 phosphorylation, exemplifying that SphK1 modulates IL-6/STAT3 signaling. Notably, we show that S1P_1_ knockdown reduced IL-6/STAT3 signaling, representing another pathway by which SphK1/S1P regulates invasion.

**Conclusions:**

Taken together, our data suggest that SphK1 sits at the hub of multiple key signaling cascades, all which have been implicated in the regulation of invasiveness, making SphK1 an attractive target for the development of HNSCC therapies.

## Background

Recurrence rates for advanced-stage head and neck squamous cell carcinoma (HNSCC) is greater than 50% [1] and the 5-year survival rate for HNSCC has not drastically improved over the last 30 years [2]. The bleak survival rate is due to late presentation, the subsequent delay of detection of lesions, invasion into loco-regional lymph nodes, a high rate of metastasis [2] and the limited availability of effective therapies.

Sphingolipids play a crucial role in cancer pathogenesis by modulating cell signal transduction pathways to influence biological outcomes such as cell senescence, differentiation, apoptosis, migration, and proliferation [3–5]. Sphingosine kinase-1 (SphK1) is an important enzyme in sphingolipid metabolism which regulates tumor growth in HNSCC. For example, SphK1 knockdown results in lower cell proliferation and smaller HNSCC tumors [6], and SphK1 inhibition increases radiation sensitivity [7].

In addition to its well-documented role in cell proliferation, SphK1 also regulates invasion. SphK1 overexpression is positively associated with invasion, invasive morphology and cell diameter in esophageal squamous carcinoma cells (ESCC) [8]. In addition, immunodeficient mice subcutaneously injected with ESCC cells overexpressing SphK1 exhibited 6-fold greater lung metastasis compared to parent cells [8]. In clinical HNSCC samples, human SphK1 expression was significantly higher compared to normal mucosa, and this was positively associated with depth of tumor invasion, metastasis, and clinical failure [9]. Furthermore, SphK1 negative staining was associated with a 6.5-year survival post-surgery, while SphK1 positive staining was associated with only a 2-year survival period post-surgery [9].

It is not known whether SphK1 is directly involved in activation of epidermal growth factor receptor (EGFR) in HNSCC. However, it is known that SphK1 expression correlates with genes downstream of the EGFR pathway (i.e., amphiregulin, integrin_α5_, epiregulin) in ESCC as demonstrated with microarray analyses [8]. Also, ESCC cells overexpressing SphK1 had greater phosphorylation of EGF, while cells transfected with siRNA against SphK1 showed reduced EGFR phosphorylation [8]. Another study showed treatment with EGFR alone increased invasion (~4×) in carcinoma of the epiglottis (PCI-37A) and inhibition of EGFR inhibited invasion by 47-fold [10]. In addition, EGFR was also shown to mediate invasion in conjunction with signal transducer and activator of transcription 3 (STAT3) in HNSCC [11]. Together these studies suggest that a relationship may exist between SphK1, EGFR and STAT3 to affect invasive ability.

The most obvious mechanism of SphK1 in mediating invasion involves sphingosine-1-phosphate (S1P)-S1P receptors interaction. After SphK increases S1P production, S1P is transported out of the cell and subsequently binds to its receptors in an autocrine and/or paracrine fashion. S1P binds to one of its 5 G-coupled protein receptors (GPCR) to activate Rac, Ras-ERK, PI3K-AKT-Rac, phospholipase C (PLC) and Rho [3]. The action of S1P on S1P receptors depends upon the activity of the associated heterotrimeric G proteins, i.e., G_i_ pathways are prolific pathways and G_12/13_ oppose proliferation and migration [12]. S1P receptor, S1P_1_, is associated with prolific heterotrimeric G_i_, which is known to stimulate migration [12,13] and it is also linked to persistent STAT3 activation, tumor growth and metastasis in colitis-associated cancer [14].

The mechanisms underlying SphK1/S1P control of invasion and its relation to EGFR and STAT3 in HNSCC remains to be clarified. Therefore, our aim was to establish the mechanistic roles of SphK1 in modulating invasion in human HNSCC. We hypothesized that SphK1 overexpression would increase invasion through mechanisms directly involving S1P_1_, EGFR and STAT3.

## Results and Discussion

### SphK1 mediates invasion in SCC-25 cells via matrix metalloproteinase (MMP)-2/9 and E-cadherin

**Figure 1:**
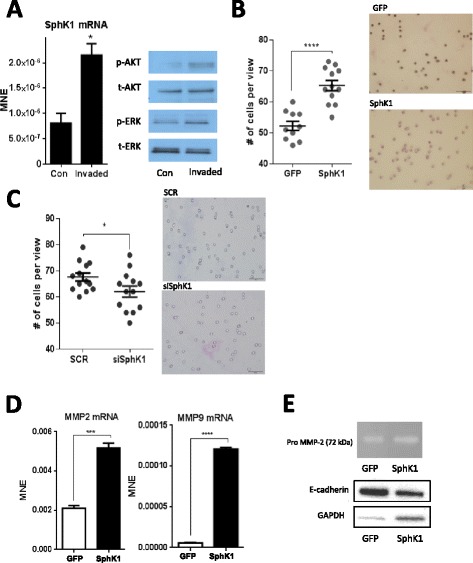
**SphK1 modulates invasion in SCC-25 cells. (A)** SCC-25 cells with an invasive phenotype have 2-fold higher mean normalized expression (MNE) of SphK1 mRNA compared to parent cells (left). SCC-25 invaded cells have greater activation of AKT (60 kDa) and ERK (42/44 kDa) compared to parent cells (immunoblot, right). **(B)** Overexpression of human SphK1 in SCC-25 cells (SphK1) significantly increased SphK1 mRNA levels compared to mock conditions (GFP) as measured with qPCR (P < 0.0001, left). SphK1 overexpressing cells displayed greater invasiveness across transwell Matrigel membrane compared to mock-transfected cells (right, invasion assay). **(C)** SphK1 transient knockdown significantly reduced the number of cells that invaded and transversed the transwell Matrigel membrane (P < 0.05, left). Image of invaded cells transected with scramble sequence (SCR) or SphK1 siRNA (siSphK1) (right). **(D)** MMP-2 and −9 mRNA were significantly increased in SphK1 overexpressing cells, (P < 0.001 and P < 0.0001, respectively, qPCR). (**E**, top) Media cultured by SphK1 overexpressing cells have slightly greater amounts of pro-MMP-2 (72 kDa) unstimulated conditions (gelatin zymography). (**E**, bottom) SphK1 overexpressing SCC-25 cells have reduced E-cadherin levels (135 kDa) compared to control conditions in basal conditions. Means ± SE are presented, *p < 0.05, ***p < 0.001, ****p < 0.0001.

To examine the role of SphK1 in invasion, first, we successfully generated an invasive cell line by recovering and propagating SCC-25 that invaded a Matrigel-coated transwell membrane. We confirmed that invasive cells had greater phosphorylation of AKT (ser473) and extracellular signal-related kinase (ERK) than parent cells on immunoblot analysis (Figure 1A, right), which are consistent with an invasive phenotype. SphK1 mRNA was compared between the invasive cells and parent cells and we found that SphK1 mRNA was 2-fold higher in the invasive cell line (Figure 1A, left), indicating that invasive cells express more SphK1 than non-invasive cells.

The next we investigated the effect of SphK1 on invasion, we employed stable transfectants and transwell cell invasion assays. We generated stable transfectants with SphK1 overexpression and siRNA in SCC-25 cells and analyzed effects of SphK1 expression in SCC-25 cells on invasion assay. We found that cells overexpressing SphK1 had significantly greater invasive ability as more cells invaded the matrigel membrane compared to mock conditions, GFP-transfected cells (P < 0.0001, Figure 1B). As expected, SphK1 knockdown reduced invasion as significantly less cells invaded the matrigel membrane as compared to SCR control (P < 0.05, Figure 1C).

Epithelial-mesenchymal transition (EMT) and degradation of the extracellular matrix (ECM) are early events in metastasis where disrupted cell-cell adhesion permits cells to invade a secondary site. Loss of cell adhesion protein E-cadherin is a hallmark of EMT [15]. Matrix metalloproteinases (MMP) also play a critical role in ECM degradation and remodeling, both necessary components of EMT. Since E-cadherin and MMPs are associated with migratory and invasive phenotypes, we next examined the role of SphK1 in regulating E-cadherin and gelatinase MMP-2 and −9.

Since E-cadherin expression is inversely correlated with lymph node metastasis in primary HNSCC [16], and we showed that SphK1 overexpression increases invasiveness, we hypothesized that SphK1 overexpression would reduce E-cadherin and increase MMP mRNA. Consistent with our hypothesis, SphK1 overexpression caused significant increases in basal MMP-2/9 mRNA levels (P < 0.001 and P < 0.0001, respectively, Figure 1D). These data indicate that SphK1 augments ECM degradation in the absence of stimulation. Together, these findings are consistent with a previous study that showed EGFR activation promotes cell migration and invasion via ERK and phosphatidylinositide 3-kiase (PI3K)/AKT-regulated MMP-9 and E-cadherin signaling pathways in HNSCC [17].

The regulation of SphK1 of MMP-2/9 was also validated using gelatin zymography. Media conditioned by SCC-25 cells overexpressing SphK1 was used to measure the amount of extracellular gelatinase MMP-2 and MMP-9 released from the cells. SphK1 overexpression slightly increased extracellular MMP-2 (pro-form) as visualized with substrate embedded gelatin zymogram assays (Figure 1E, upper panel). While pro-forms of MMP are generally regarded as inactive, they have been previously used as markers of enhanced synthesis of MMP [18,19].

SphK1 overexpression alone in the absence of stimulation reduced E-cadherin protein levels as measured with immunoblotting (Figure 1E, lower panel). These data indicate that SphK1 overexpression supports EMT initiation via with a reduction of E-cadherin and induction in MMP-2/9 mRNA. SphK1 overexpression increases invasiveness, and is paralleled with changes associated with EMT.

### SphK1 regulates EGFR, IL-6 and STAT3

**Figure 2:**
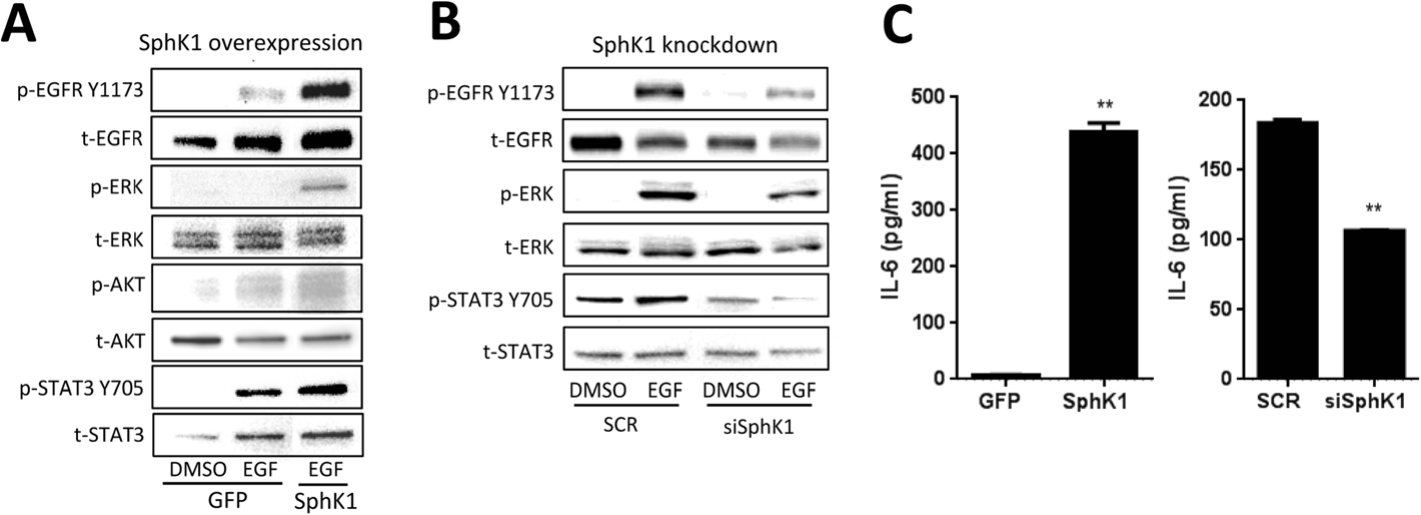
**SphK1 regulates EGFR and STAT3. (A)** p-EGFR (Y1173), p-STAT3 (Y705), p-ERK, and p-AKT induced by EGF are greater in SphK1 overexpressing cells compared to mock conditions (Immunoblot). **(B)** SphK1 transient knockdown reduced EGF-induced EGFR, STAT3, and ERK phosphorylation (Immunoblot). **(C)** IL-6 levels are significantly greater in media conditioned by SphK1 overexpressing cells versus mock conditions (P < 0.01, ELISA, left). SphK1 knockdown significantly reduced IL-6 concentration in conditioned media as measured with ELISA (P < 0.01, right). Means ± SE are presented, **p < 0.01, ***p < 0.001.

Next we show SphK1 expression mediates signal transduction markers associated with invasion and metastasis. As expected, EGF-induced phosphorylation of EGFR was greater in SphK1 overexpressing cells compared to mock conditions (Figure 2A). This is consistent with a previous report documenting that SphK1 overexpression increased EGFR phosphorylation in ESCC [8]. In addition, SphK1 overexpression increased EGF-induced phosphorylation of ERK with a slight increase in p-AKT when compared to mock conditions, consistent with an invasive phenotype (Figure 2A). Conversely, SphK1 knockdown reduced both p-EGFR and p-ERK (Figure 2B), consistent with a previous report documenting that SphK1 knockdown reduces EGFR phosphorylation [8].

SphK1 overexpression also increased EGF-induced p-STAT3 (Figure 2A) and this was paralleled with significantly elevated extracellular IL-6 levels as measured with an ELISA assay (P < 0.01, Figure 2C, left panel). Conversely, when SphK1 was knocked down, p-STAT3 was down regulated, suggesting that SphK1 may directly regulate STAT3 (Figure 2C). SphK1 knockdown also caused a 40% reduction in extracellular IL-6 concentrations as measured in conditioned media (P < 0.01, Figure 2C right panel). Since IL-6 levels drive STAT3 activation in HNSCC [20], perturbation of p-STAT3 may be due to SphK1-mediated changes in extracellular IL-6 concentrations.

EGFR variant III (vIII), a constitutively active form of EGFR, has been shown to mediate HNSCC invasion and cell proliferation through increased STAT3 activation [11]. Since we have shown that SphK1 regulates p-EGFR, these changes in STAT3 may be mediated by SphK1-induced alterations in EGFR. Alternatively, SphK1 may directly regulate IL-6 levels, and the fluctuation in IL-6 levels may alter downstream STAT3 signaling.

### S1P_1_ regulates invasion

**Figure 3:**
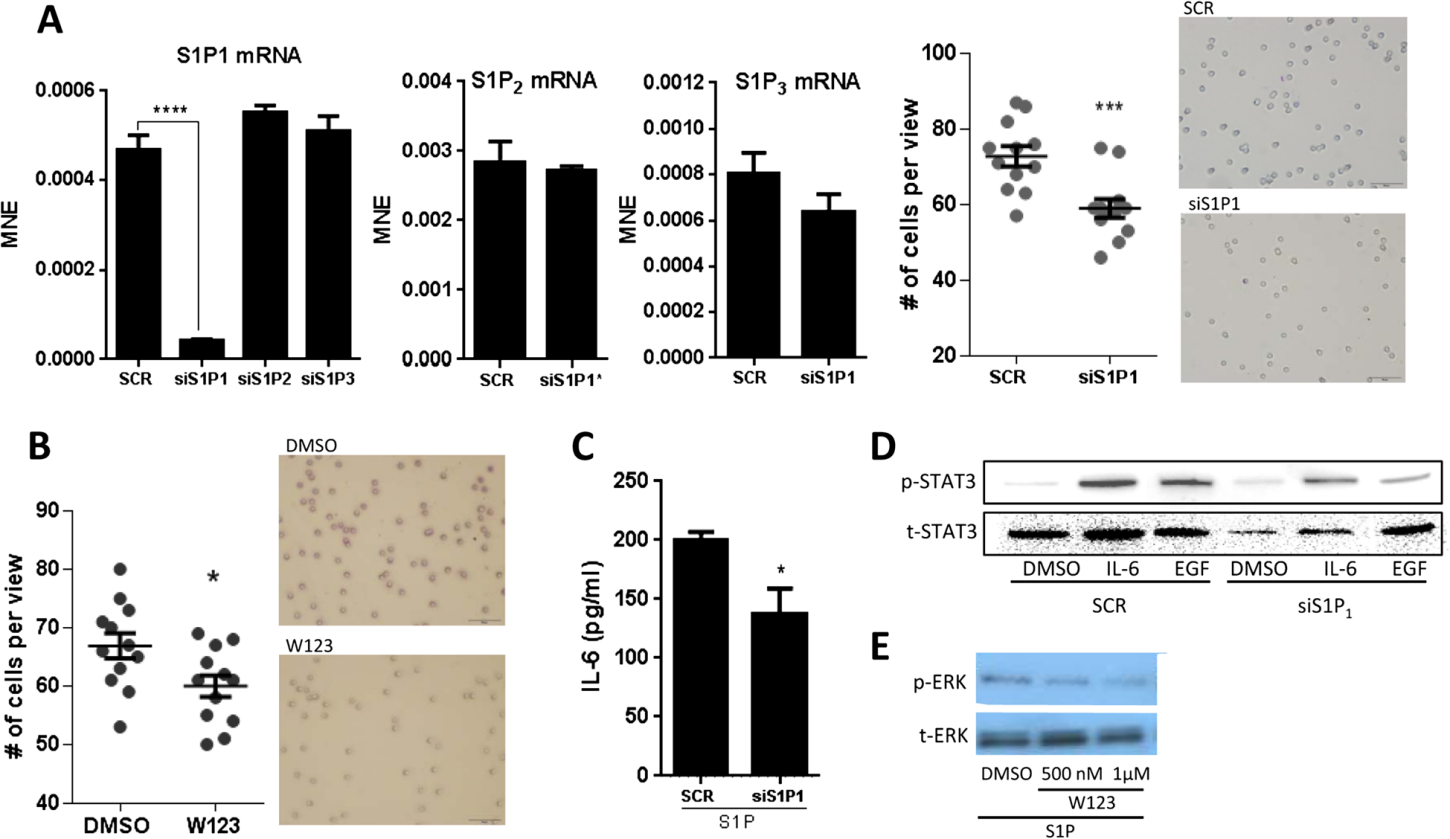
**S1P**_**1**_ **regulates invasion in SCC-25. (A)** siRNA targeted knockdown of S1P_1_ was specific to S1P_1_ mRNA (qPCR, left), did not affect S1P_2_ or S1P_3_ mRNA (qPCR, middle panels). S1P_1_ down-regulation significantly reduced invasion (images of invaded cells, P < 0.001, far right). **(B)** Treatment with W123, an S1P_1_ inhibitor (1 μM), significantly reduced invasion (invasion assay, P < 0.05). **(C)** S1P_1_ knockdown significantly reduced IL-6 levels in conditioned media under S1P stimulation (P < 0.05, ELISA). **(D)** S1P_1_ knockdown reduced IL-6- and EGF-induced increases in p-STAT3 (Immunoblot). **(E)** Treatment with W123 (500 nM, 1 μM) reduced ERK phosphorylation in a dose dependent manner under S1P stimulation (Immunoblot). S1P was dissolved in ethanol. Means ± SE are presented, *p < 0.05, ***p < 0.001, ***p < 0.001.

Next, we investigated whether S1P_1_ is associated with SphK1-related invasion in HNSCC. S1P_1_ knockdown was achieved using siRNA specific to the receptor and we confirmed that S1P_1_ knockdown significantly reduced S1P_1_ mRNA levels (P < 0.0001), but did not affect other receptors, mainly S1P_2_ and S1P_3_ (Figure 3A). Invasion assays showed that S1P_1_ down regulation by siRNA and inhibition with inhibitor W123 (1 μM) significantly reduced invasion of SCC-25 cells overexpressing SphK1 (Figure 3B, P < 0.001 and P < 0.05, respectively), indicating that S1P_1_ is associated with invasion due to S1P_1_ is coupled to G_i_ that promotes migration. These observations indicate that one mechanism of action by which SphK1 regulates invasion is through S1P-S1P_1_ interaction.

We found that S1P_1_ knockdown reduced extracellular IL-6 concentrations in media conditioned by cells treated with S1P (Figure 3C). S1P_1_ knockdown also reduced IL-6 induced phosphorylation of STAT3 (Figure 3D), indicating that S1P_1_ is required for full activation of STAT3. Here, S1P_1_ knockdown reduced STAT3 activation, leading to reduced extracellular IL-6 concentrations. This suggests that not only does IL-6 drive STAT3 activation as previously documented [20], but S1P_1_ knockdown also exerts control over STAT3 activation and affects downstream signaling to dampen IL-6 production and release.

In addition, S1P_1_ knockdown reduced EGF-induced phosphorylation of STAT3 (Figure 3D). Inhibition of S1P_1_ with increasing dosages of W123 (500 nM, 1 μM) caused dose-dependent decreases in p-ERK when stimulated with S1P (Figure 3E), confirming that S1P_1_ is an important regulator of ERK activation. Both S1P_1_ and EGFR regulate downstream ERK signaling. However, it appears that S1P_1_ knockdown has the capacity to abrogate EGF-induced ERK activation, implying S1P_1_ is a highly influential regulator of ERK.

The key finding is that S1P_1_ inhibition resulted in reduced invasive ability *in vitro* and this coincided with reduced EGF-induced STAT3 activation and IL-6 levels. S1P stimulation has previously been shown to induce invasion and migration through induction of MMP-9 in human breast epithelial cells (MCF10A) [18]. Thus, it is expected that perturbation of S1P_1_ would affect invasion. In fact, a poster presentation demonstrated that S1P_1_ expression levels in primary oral cavity SCC from patients with cervical lymph node metastasis were significantly higher compared to those without metastasis [21]. This poster also stated that 20 out of 30 metastatic lymph node samples had cancer cells with higher S1P_1_ expression compared to cells in primary tumors, highlighting the importance of S1P_1_ in invasion and metastasis.

Notably, we observed that S1P_1_ knockdown reduced IL-6 and EGF-induced activation of STAT3. The association among IL-6, S1P_1_ and STAT3 in the current study is similar to the relationship observed in colitis-associated cancer where reduced SphK1 and S1P_1_ expression abrogated IL-6/STAT3 signaling, halting the development of colitis-associated cancer [22].

One other study showed that SphK1 positively impacted cellular invasion via upregulation of MMP-2/9 via p-ERK in colon cancer [23]. However, this study focused only on SphK1 and not its receptor S1P_1_. Therefore, it is unclear whether the upregulation of MMP is mainly due to increased signaling activity of the SphK1/S1P1 axis or the SphK1/EGFR axis.

We also show SphK1 is central to the regulation of EGFR and IL-6/STAT3, highly influential and significant pathways which regulate invasiveness. EGFR is overexpressed in 40-90% of HNSCC [24–26], and causes potent stimulation of invasion and metastasis [17]. STAT3 regulation is another potent regulator of invasion [27], where STAT3 binds directly to the MMP-2 promoter with a high affinity [28] to directly mediate tumor invasion and metastasis. Thus, the ability of SphK1 and S1P_1_ to regulate both STAT3 and EGFR indicate that they are central to invasive control.

**Figure 4:**
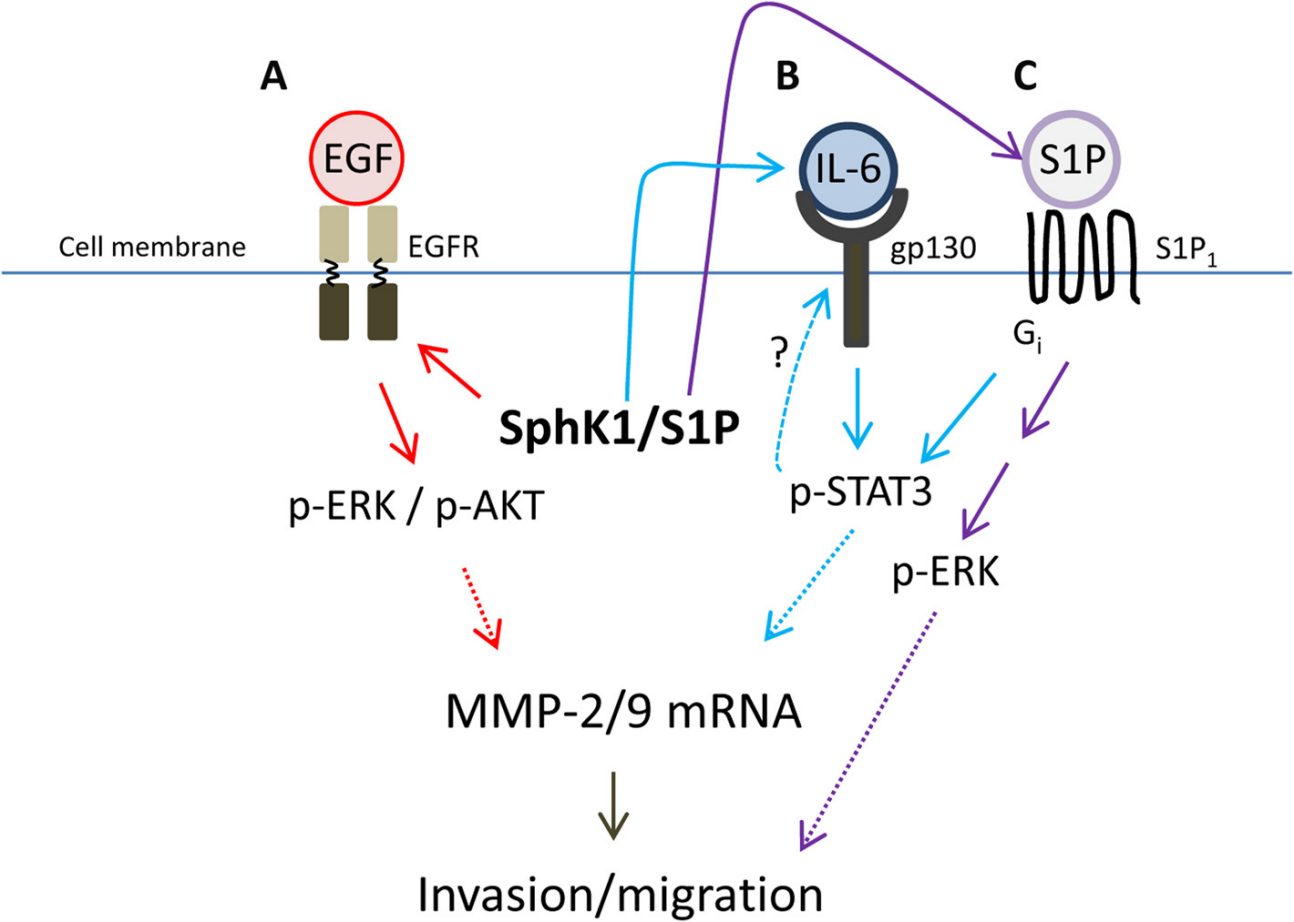
**Role of SphK1 in regulating invasion in HNSCC. (A)** SphK1/S1P regulated p-EGFR such that SphK1 overexpression increased p-EGFR and p-ERK and SphK1 knockdown reduced EGFR and ERK phosphorylation. This regulation of p-EGFR putatively affected invasiveness and motility in SCC-25 cells via MMP-2/9 and E-cadherin. **(B)** SphK1 overexpression increases IL-6 concentration and p-STAT3, while SphK1 knockdown reduces IL-6 and STAT3 activation. **(C)** S1P_1_ knockdown reduced invasion in vitro. This may be due to S1P_1_ regulation of STAT3 as S1P_1_ knockdown reduced extracellular IL-6 levels and IL-6- and EGF-induced STAT3 activation.

We provide evidence of possible mechanisms by which SphK1 regulates invasion (as depicted in Figure 4). For example, SphK1 mediates EGFR, a potent stimulator of invasion and metastasis. EGFR activation promotes cell migration and invasion in HNSCC through MMP-9-mediated degradation of E-cadherin, subsequent ERK activation and AKT/PI3K signaling [17]. In our model, we also found that SphK1 overexpression coincides with increased MMP-2/9 mRNA and decreased E-cadherin protein levels. A previous report indicates that SphK1 is required for MMP-2/9 production and this response is dependent upon by ERK activation in colon cancer cells [23]. Thus, it is not unlikely that SphK1 also regulates EMT in HNSCC.

SphK1 overexpression promoted increased IL-6 concentrations, and EGF-induced EGFR and STAT3 phosphorylation. It is known that EGFR and STAT3 phosphorylation promote invasion through alterations in MMP-2/9 and E-cadherin [17,28,29]. Thus, it appears that SphK1 overexpression increased EGFR and STAT3 activation, which in turn upregulated MMP mRNA and reduced E-cadherin, resulting in increased motility. SphK1 knockdown also decreased IL-6 levels and EGFR and STAT3 activation. Notably, SphK1 knockdown reduced STAT3 activation in the absence of stimulation, indicating SphK1 regulates STAT3 under basal conditions. These data are in consensus with previous reports indicating that SphK1 is positively associated with metastasis and invasion in HNSCC [6–8].

Based upon the fact that sphingolipids largely exist in cellular membranes and provide the structure of membrane-associated lipid rafts and regulate caveolar-mediated endocytosis, we cannot exclude the possibility that the effect of SphK1 on invasion may involve low-density lipoprotein receptor-related protein-1 (LRP-1). The protein encoded by LRP-1 regulates intracellular signaling, lipid homeostasis and clearance of apoptotic cells. Notably, LRP-1 are transiently associated with lipid rafts/caveloe [30], is an upstream regulator of MMP-2/9 [31], and signal mainly through ERK to mediate adhesive complex turnover leading to migration and invasion [32]. The effects of LRP-1 parallel our findings and thus, we cannot rule out the possibility that perturbation of SphK1 (and associated sphingolipids) dysregulate microdomain structure and lipid rafts which fuels a cascade of events leading to invasion.

Although we focused mainly on SphK1 and S1P_1_, other sphingolipid-related players may have been involved (namely SphK2, ceramide, and receptors S1P_2,3,4,5_). The importance of SphK2 in HNSCC is currently unknown. However, in human esophageal adenocarcinoma (OE33) SphK2 affected migration but not invasion (which requires degradation of matrigel) [33]. SphK1 affected migration to a larger extent as compared to SphK2, suggesting SphK1 may be more important in determining invasive ability. Still, further research is needed to examine the roles of these sphingolipid-related mediators in regulating invasion in HNSCC.

## Conclusions

Our data indicate that knockdown of S1P_1_ significantly reduces IL-6/STAT3 signaling; SphK1 positively regulates EGFR and STAT3 signaling to increase invasive ability. Since SphK1/S1P_1_ represents a point where multiple signal pathways converge to regulate invasion, and SphK1 is expressed in all stages of HNSCC, targeting SphK1/S1P_1_ may be a successful route in the development of HNSCC therapy strategies.

## Methods

### Cell culture and treatment

Human tongue squamous cell carcinoma (SCC-25 cells) was obtained from the Harvard Skin Disease Research Center (Boston, MA) and was maintained at 37%/5% CO_2_. Cells were cultured in DMEM/F12 media (ATCC, Manassas, VA) supplemented with 10% fetal bovine serum (FBS, Invitrogen, Grand Island, NY) and 100 U/ml penicillin-100 μg/ml streptomycin (Invitrogen). Cells were serum starved 24 hours prior to treatment with epidermal growth factor (EGF, 50 ng/ml, 15 minutes) or interleukin-6 (IL-6, 30 ng/ml, 30 minutes). W123 (1 μM, Cayman Chemical, Boston, MA) were applied for 2 hours prior to stimulation.

### Preparation of SphK1 plasmid and stable transfectant

LR clonase enzyme (Invitrogen) was used to insert entry vector, purified pDONR223-SPHK1 (human Sphingosine Kinase-1, plasmid 23704, Addgene, Cambridge MA), into destination vector pcDNA-DEST40 Vector (12274-015, Invitrogen). One Shot TOP10 Chemically Competent E. coli (C4040-03, Invitrogen) was transformed with SphK1-DEST-40 plasmid DNA. A single colony was recovered and propagated overnight and the plasmid was purified using a HiSpeed Plasmid Purification Midi Kit (Qiagen, Valencia, CA).

SCC-25 cells were then transfected using jetPRIME (PolyPlus transfection, New York) according to manufacturer’s guidelines. Briefly, 4 μl of jetPRIME solution was added to 2 μg of DNA in jetPRIME buffer and incubated for 10 minutes at room temperature. The transfection solution was added to cells at 60-80% confluency; transfection media was removed 4 hour post-transfection. Geneticin (G418, Life Technologies, Carlsbad, CA) selection began 2–3 days later. SphK1 mRNA level was measured to confirm successful transfection. Green fluorescence protein (GFP) was used as a mock vector (pENTRY-GFP, Plasmid 15301, Addgene).

### Small-interfering (siRNA) transfection

Transient knockdown of SphK1 with siRNA was performed as previously described [34]. Briefly, siRNA targeting human SphK1 (Qiagen) was diluted into Opti-MEM (Invitrogen) and then incubated with transfection reagent, Lipofectamine RNAiMax (Invitrogen) prior to plating SCC-25 cells (final siRNA concentration10 nM). Cells were incubated for 24 hours with the transfection reagent. A scrambled sequence with no known homology to mammalian genes was used as a negative control (Qiagen).

### Quantitative real-time RT-PCR

RNA extraction was performed according to manufacturer’s guidelines (RNeasy mini kit, Qiagen). cDNA was synthesized using qScript cDNA SuperMix (Quanta Biosciences, Gaithersburg, MD). Real-time PCR was performed with MyiQ2 Two-Color Real-Time PCR Detection System (Bio-Rad Laboratories, Hercules, CA) as previously described [34]. The standard real-time PCR reaction volume was 20 μl, and consisted of 10 μl of PerfeCTa SYBR Green FastMix (Quanta Biosciences), 7 μl RNAse-free H_2_O, 1 μl forward primer, 1 μl reverse primer and 1 μl cDNA. Initial step at 95% for 30 seconds and 40 cycles consisted of 30 seconds of melting at 95%, followed by 30 seconds of annealing/extension at 60%. All reactions were performed in triplicate. Threshold cycle (C_T_) analysis for all samples was set at 0.15 relative fluorescence units. The data are expressed as mean normalized expression (MNE). MNE is directly proportional to the amount of RNA of the target gene relative to the amount of RNA of the reference gene GAPDH.

The following primers were used for qPCR amplification: Human SphK1 Forward 5′- AGGCTGAAATCTCCTTCACGC-3′ and reverse 5′-GTCTCCAGACATGACCACCAG-3′; Human GAPDH Forward 5′-AGGGCTGCTTTTAACTCTGGT-3′ and reverse 5′-CCCCACTTGATTTTGGAGGGA-3′; Human MMP-2 Forward 5′-CCCACTGCGGTTTTCTCGAAT-3′ and reverse 5′-CAAAGGGGTATCCATCGCCAT-3′; Human MMP-9 Forward 5′-TCGTGGTTCCAACTCGGTTT-3′ and reverse 5′-GGTTTCCCATCAGCATTGCC-3′; Human S1P_1_ Forward 5′-GCTGGGTCATCTCCCTCAT-3′ and reverse 5′-GCAGTTCCAGCCCATGAT-3′; Human S1P_2_ Forward 5′-CCAACAAGGTCCAGGAACAC-3′ and reverse 5′-GCAACAGAGGATGACGATGA-3′; Human S1P_3_ Forward 5′- TCAGGGAGGGCAGTATGTTC-3′ and reverse 5′-CCAGTAAGCTGCAGGTGGA-3′.

### Invasion assay

An *in vitro* invasion assay was performed using a 24-well format in quadruplicate according to manufacturer’s guidelines (BD BioCoat Matrigel Invasion chamber, BD Biosciences, San Jose, CA). Briefly, after hydration of membranes, stable SCC-25 transfectants overexpressing SphK1 were seeded (0.35 × 10^5^ cell/well) in the presence of FBS, G418 and EGF (50 ng/ml). Twenty-two hours later, non-invaded cells were removed and invaded cells were fixed and stained (Diff-Quik, VWR, Radnor, PA). Membranes were carefully removed and mounted on glass slides with immersion oil. Three fields per membrane (1 center and 2 peripheral views) were visualized with a light microscope (Olympus BX51) and images were captured with a camera (Olympus DP72, 400× magnification). The number of invaded cells per view was counted and recorded for each image. The mean number of invaded cells was used to compare invasive ability between conditions.

#### Establishment of invasive phenotype

An invasive cell line using SCC-25 was generated using a similar method as outlined above, with a few deviations. 3 × 10^5^ cells were seeded into each well in complete media (DMEM/F12 supplemented with 10% FBS and 1% penicillin/streptomycin) in a 6-well BD BioCoat Matrigel Invasion system. 22 hrs later, invaded cells were trypsinized from the membrane, plated in a single well of a 6-well plate, and propagated.

#### Reverse transfection in invasion assay

SphK1 overexpressing SCC-25 cells were used for all invasion assays. Knockdown of S1PR with siRNA was performed in the upper compartment of the invasion chamber. For reverse transfection, siRNA (EDG-1/S1P_1_, EDG-5/S1P2, EDG-3/S1P3) was used at a final concentration of 10 nM (Santa Cruz Biotechnology, Dallas, TX). siRNA were diluted into Opti-MEM (Invitrogen) and then incubated with transfection reagent Lipofectamine RNAiMax (Invitrogen) before cells were seeded onto the Matrigel membrane (0.35 × 10^5^ cell/well). Experiments were conducted in quadruplicate. Cells were incubated for 24 hours with the transfection reagent. A scrambled sequence with no known homology was used as a negative control (Qiagen).

### Substrate Embedded Gelatin Zymography Assay

Cells were seeded at a density of 3.5 × 10^5^ per well in DMEM/F12. Twenty-four hours later, cells were serum starved in 1 ml of media for an additional 24 hours prior to assay. Conditioned media was collected, centrifuged to remove cell debris and supernatant was collected in a clean tube. Conditioned media was mixed 1:1 with 2× native sample buffer (100 mM Tris-Cl (pH 6.8), 4% SDS, 0.2% Bromophenol blue, 20% glycerol) and separated on a 10% SDS-polyacrylamide gel containing 0.1% gelatin under non-reducing conditions. After electrophoresis, the gel was incubated with 2.5% (v/v) Triton X-100 at room temperature for 2 hours, gelatinase buffer (50 mM Tris–HCl, pH 7.5, 50 mM NaCl, and 10 mM CaCl_2_) at 37% with gentle shaking for 12–24 hours and then stained with 0.25% (g/ml) Coomassie brilliant blue R-250 at room temperature for 4–6 hours. Gels were then de-stained with acetic acid/methanol/deionized water (10%/10%/80%) until clear bands appeared on the gel. Areas showing enzyme activity show up as regions of negative staining.

### Immunoblots

Cells were washed, lysed and collected (5 M NaCl, 1 M Tris–HCl (pH 7.5), 1 M MgCl_2_, 0.1 M EGTA, plustprotease/phosphatase inhibitors) and 20–25 μg of protein was resolved on a 4–20% Tris–HCl gel (Bio-Rad, Hercules, CA). Proteins were transferred to polyvinyl difluoride membranes, blocked with I-block (Life Technologies, Carlsbad, CA), and incubated with specific primary antibodies (all antibodies were purchased from Cell Signaling, Danvers, MA). Membranes were incubated with horseradish peroxidase-linked secondary antibodies (Santa Cruz) and signals were visualized using Pierce enhanced chemiluminescence Western blotting substrate (Thermo Scientific, Waltham, MA) and film or Clarity Western enhanced chemiluminescence substrate (Bio-Rad) and a digital darkroom (FluorChem M system, ProteinSimple, Santa Clara, CA).

### IL-6 ELISA

Human IL-6 was measured in conditioned media using a commercially available ELISA kit (BM213 INST, eBioscience, San Diego, CA). The protocol was followed according to manufacturer’s guidelines. Briefly, 3 × 10^5^ cells were seeded in a 6-well plate in 1 ml of media for 24 hours. Media was collected, centrifuged and pure supernatant was analyzed. Media was diluted 1:5 prior to assay and IL-6 concentration was measured in duplicate. Samples were applied to the plate incubated for 3 hours with shaking (room temperature), washed, and 3,3′,5,5′-Tetramethylbenzidine (TMB) substrate was added. The reaction was stopped 5 minutes later and absorbance was read at 450 nM (with a secondary wave length of 620 nM). The standard curve ranging from 0 to 200 pg/ml was used to calculate concentration based upon absorbance and optical density; concentrations were multiplied by 5 to account for the dilution.

### Statistics

One-way ANOVAs (and Tukey *post hoc* comparisons) were used to compare groups of 3 or more and unpaired t-tests (two-tailed) were used for analyses involving 2 groups (Prism Graphpad, La Jolla, CA). Statistical significance was set at an α-level of 0.05. Means ± SEM are presented.

## Abbreviations

ECM: Extracellular matrix
EGF: Epidermal growth factor
EGFR: Epidermal growth factor receptor
EMT: Epithelial-mesenchymal transition, ESCC, esophageal squamous cell carcinoma
HNSCC: Head and neck squamous cell carcinoma
IL-6: Interleukin-6
LRP-1: Low-density lipoprotein receptor-related protein-1
MMP: Matrix metalloproteinase
NFkB: NFkappaB
S1P: Sphingosine 1-phosphate
SphK1: Sphingosine kinase-1
SphK2: Sphingosine kinase-2
STAT3: Signal transducer and activator of transcription 3
S1PR: Sphingosine 1-phosphate receptor
